# The Prevalence of Human Papillomavirus between the Neonates and Their Mothers

**DOI:** 10.1155/2015/126417

**Published:** 2015-12-02

**Authors:** Mariusz Skoczyński, Anna Goździcka-Józefiak, Anna Kwaśniewska

**Affiliations:** ^1^Department of Obstetrics and Pathology of Pregnancy, Medical University of Lublin, 20059 Lublin, Poland; ^2^Department of Molecular Virology, Institute of Experimental Biology, Adam Mickiewicz University, 61712 Poznań, Poland

## Abstract

The impact of human papillomavirus (HPV) infection on pregnancy is a major problem of medicine. The transmission of the virus from mother to fetus is a process yet unresolved. The immune response and changed hormonal status of pregnant women might facilitate infection. A research on the prevalence of HPV infection was conducted at the Clinic of Obstetrics, Medical University of Lublin (Poland). The studied group included 152 randomly selected women. The material was tested for the presence of HPV DNA by means of polymerase chain reaction (PCR). The aim of the research was to assess the relation between HPV infections detected in the buccal smears of the neonates and the incidence of such infections in the cervical/buccal smears of their mothers. In the group of 152 infants HPV was found in 16 (10.53%). Among the cervical/buccal smears, HPV was isolated, respectively, in 24 (15.79%) and in 19 (12.5%) pregnant women. Statistically significant differences in the prevalence of HPV swabs from the newborns and the cervical/buccal smears of their mothers were found (*p* < 0.001). The identification of mothers in whose buccal smears HPV was detected can help develop a group of children who run a relatively significant risk of being infected.

## 1. Introduction

The influence of human papillomavirus infection on pregnancy remains an unresolved matter. HPV infection is commonly included in the group of sexually transmitted diseases (STDs) and is one of the most frequently transmitted among them [1, 2]. The influence of the high-risk HPV virus on cancerous processes is a commonly accepted hypothesis [3]. The relation between HPV infection and the development of RRP (Recurrent Respiratory Papillomatosis) in newborns cannot be omitted. According to the various reports 30% to 50% of the clinical signs of the infection in mucous membrane of the upper respiratory tract caused by human papillomavirus can be associated with mother's HPV infection [4–6]. The estimated risk ratio of infection among young women is 80%, although the majority of infections are asymptomatic and show a tendency to cure spontaneously [7, 8]. It should, however, be underlined that women aged between 20 and 35 not only are most prone to getting infected with HPV but also are most sexually active, get pregnant, and have and raise children. The possibility of HPV transmission from mother to fetus occurring during pregnancy and perinatal period has been postulated. It is, however, still undetermined and sparks heated debate. Therefore, identifying the risk factors and mechanisms of transmission of the virus, including the impact of hormonal and immunological changes during pregnancy, is of vital importance. Taking into consideration the current epidemiological data it can be concluded that HPV transmission from mother to newborn is possible before, during, and after birth [9–11]. It confirms the importance of the transmission of the virus from mother to her baby. The presented study aims to compare the frequency of HPV infection in pregnant women and their newborns in different places. An attempt to find correlation between mothers' and newborns' infections was made to select a group of children prone to HPV-related entities. Apart from the cytological examination of cervix, the smear of oral mucosa both from mothers and their babies used for the identification and prophylaxis of HPV infection could be relevant in clinical practice.

## 2. Materials and Methods

This study was conducted between March 2009 and June 2011 and included 152 pregnant women who delivered at the Clinic of Obstetrics and Pathology of Pregnancy, Medical University of Lublin (Poland). The studied group included random obstetric population recruited prior to the delivery. The routine medical examination was followed by previous history of HPV-related genital diseases and treatments. The study had the following inclusion criteria: (1) singleton pregnancy, (2) lack of clinical symptoms suggesting the possibility of HPV infection, (3) normal cervical smear, and (4) term delivery. The number of women who refused was 17. The exclusion criteria included (1) history of HPV infection (*n* = 7), (2) abnormal cervical smear (*n* = 4), and (3) multiple pregnancy (*n* = 6). The subjects gave their written, informed consent before the start of any procedure. The protocol of this study was approved by the Local Bioethical Committee at the Medical University of Lublin. Prior to delivery, smears were obtained from the cervix and buccal mucosa of each pregnant woman. The cervical HPV DNA specimen was collected with a cytobrush inserted into the vagina. The cervix and ectocervix were swabbed using a circular motion. The oral specimens were collected during the same time. Immediately after the birth, the neonatal oral mucosa smears were obtained in similar proceedings with a cytobrush. All samples were collected by the same, properly trained person. The material was placed in sterile tubes for HPV DNA testing (Eurotubo; Deltalab, Spain), frozen, and stored at 70°C until further analyses. The material was tested for the presence of HPV DNA by means of PCR method. Identification of HPV types was performed using consensus PCR primers for L1:MY09: 5′-CGTCCMARRGGAWACTGATC-3′ and MY11: 5′-GCMCAAGGWCATAAYAATGG-3′, where M = A + C, R = A + G, W = A + T, and Y = C + T. This set of primers amplifies DNA from at least 33 different HPV genotypes. HPV types 16 and/or 18 were identified using the following type specific PCR primers: HPV16/L1A/HPV16/L1B, 5′-GCCTGTGTAGGTGTTGAGGT-3′, and 5′-TGGATTTACTCCAACATTGG-3′ product size: 264 bp; HPV18/L1A/HPV18/L1B, 5′-GTGGACCAGCAAATACAGGA-3′, and 5′-TGCAACGACCACGTGTTGGA-3′, product site: 162 bp; HPV18ME12/HPV18ME50/E6, 5′-CACGGCGACCCTACAAGCTACCTG-3′, and 5′-TGCAGCACGAATTGGCACTGGCCTC-3′, product site: 404 bp. The reaction was performed according to Tucker et al. [12]. The total volume of 10 *μ*L of PCR mixture contained 1 *μ*M of primers, 200 *μ*M of deoxynucleotide triphosphates, 1x PCR buffer (0.1 M Tris-HCl pH 8.8, 0.5 M KCl, 0.015 M MgCl_2_, 1% Triton X-100), the investigated DNA (10 ng/*μ*L), and Tag polymerase at a final concentration of 40 U/mL. After preliminary denaturation (15 min at 94°C), samples were amplified for 31 cycles in a thermal cycler. Each cycle consisted of the following steps: denaturation at 94°C for 30 seconds and annealing at 59°C for the same amount of time, followed by primer extension at 72°C for 1 min. In the last PCR cycle, the stage of complementary DNA synthesis at 72°C was extended to 420 seconds. PCR products were analysed using agarose gel electrophoresis in the presence of pBluescript DNA digested with HindI. The presence of HPV DNA was additionally verified, and HPV genotypes were identified by direct sequencing from CR reaction tube (without purification from MY09 and MY11 oligonucleotides). The results of sequencing were analysed using the BLAST database (http://blast.ncbi.nlm.nih.gov/).

### 2.1. Statistical Analysis

Normal distribution of continuous variables was verified with the Kolmogorov-Smirnoff test. Statistical characteristics of these variables were presented as arithmetic means and standard deviations (SD), and their intergroup comparisons were performed with Student's *t*-test for independent variables. All calculations were carried out with Statistica 10 (Stat Soft, Tulsa, OK, USA) package, with the level of significance set at *p* ≤ 0.05.

## 3. Results and Discussion

### 3.1. Incidence of HPV Infection in the Oral Mucosa of Mother and Her Child

As the result of the conducted research HPV DNA was isolated in 12.5% (19/152) of swabs from the oral mucosa of the mothers and in 10.53% (16/152) of the children. In the group of nineteen mothers with HPV infection we found 8 (42.11%) HPV-positive children and 11 (57.89%) HPV-negative children. In the mothers group without HPV infection 133/152 (87.5%) we observed 8/133 (6.02%) HPV (+) newborns and 125/133 (93.98%) HPV (−) newborns. A statistically significant difference in the prevalence of HPV DNA in the swabs from the oral mucosa of the newborns and the swabs from the oral mucosa of their mothers was found (*p* < 0.001). There is an eleven times greater relative risk (OR = 11.363 CI: 3.536–36.509) of discovering HPV DNA in buccal smears of newborn of a HPV-positive mother than of the mother with negative results of HPV. The correlation of the frequency of HPV infections was presented in Table 1.

The idea of HPV transmission from mother to child was first introduced by Sedlacek et al. [13] in 1989. Depending on the population tested, the place where the tested material is obtained, and methods used to identify HPV DNA the frequency of HPV transmission varies from 4% to 80% [14, 15]. According to Bandyopadhyay et al. such a wide range of gathered results is also a proof of temporary contamination with the infected material [16]. Cason et al. suggested that buccal smears provide better material for analysis of HPV DNA incidence than respiratory discharge samples [17]. However, in contrast to our data, Eppel et al. presented approximately two times higher results in their PCR study. The prevalence of HPV DNA in specimens from cervical smears of asymptomatic pregnant women was found in 24, 6% (44/179) [18]. Such a confusing difference may be due to several confounders, including study design, choice of detection methods, and differences in risk factors for HPV infection. Another research was conducted in 2005 in Finland when the first prospective tests of HPV infection incidence were ordered among family members. This study analysed the relationship between the presences of HPV infection in family environment. The team of researchers led by Rintala focused on a group of 76 families and recognized HPV infection in 8% of buccal smears of mothers and 10% of buccal smears of their neonates. The results of our research conducted on a study group twice the size show similarities [19].

### 3.2. Incidence of HPV Infection in the Oral Mucosa of Child and the Cervical Smears of Mother

In the studied group (*n* = 152) HPV DNA was isolated in 24 (15.79%) swabs from the mothers' cervix, and in 16 (10.52%) from oral mucosa of newborns. Out of 24 HPV-positive mothers, HPV DNA was isolated in 11 (45.83%) of their children. In group of 24 HPV-positive mothers we found 13 (54.17%) HPV-negative children. In the mothers group without HPV infection 128/152 (84.21%) we observed 5/128 (3.91%) HPV (+) newborns and 123/128 (96.09%) HPV (−) newborns. A statistically significant difference in the prevalence of HPV DNA in the swabs from the oral mucosa of the newborns and the swabs from the cervix of their mothers was found (*p* < 0.001). Based on the analysis of the results the relative risk of HPV DNA existing in swabs from the oral mucosa of the children whose mothers were tested positive for HPV in cervical smears is almost 21 times greater than in children whose mothers were diagnosed as healthy (OR = 20.815, CI: 6.197–69.914). The correlation between HPV infection in oral mucosa of children and the cervix of mother was presented in Table 2.

Similar results were obtained by Rice et al. [20] as HPV DNA was found in 30% of the children whose mothers were infected. They concluded that mothers with high HPV-16 viral loads in their genital tracts are usually the source of newborns infections. How many newborns are infected remains a vital question. It was suggested by the authors that there is a correlation between the type of virus and its transmissive abilities. They recognized that not enough attention was paid to show that at least 20% of healthy children present HPV infection connected with perinatal time. The same observation was supported by Puranen et al. [15]. They concluded that perinatal acquired HPV infection might persist in the oral cavity for years without any significant clinical lesions and infected mother can transmit human papillomavirus to her child. In this study HPV DNA was found in 31 of the 98 (31.6%) mothers' oral scrapings. Our study revealed 45.83% neonatal prevalence of HPV, in children whose mothers were tested HPV positive in cervical smears at delivery. Very similar data was presented by Sedlacek et al. [13]. They demonstrated HPV DNA in the oral cavity of 47.8% (11/23) of the neonates delivered vaginally from mothers in whose cervical cells HPV DNA was detected. These results are also consistent with those reported in Puranen et al. study [15]. It has been suggested that neonates could acquire HPV infection as they pass through an infected birth canal [13, 15, 20].

### 3.3. HPV Infection Incidence in Women Giving Birth

HPV DNA was identified in 29 women giving birth which was 19.08% of the total population (*n* = 152). Among the swabs of the cervical smears HPV DNA was isolated in 24/152 (15.79%) women and among the swabs from the oral mucosa HPV DNA was observed in 19/152 (12.5%). Both variables are separable and analysed as such. Simultaneous HPV infection in both swabs from the oral mucosa and the cervix was found in 14/152 (9.21%) of the total population. No HPV DNA was found in any of the assessed swabs taken from 123/152 (80.92%) women giving birth. As a result of the research a statistically significant difference in HPV DNA incidence in cervical smears in relation to HPV DNA occurrence in buccal smears of women was found (*p* < 0.001). Out of 19 women in whose swabs from the oral mucosa HPV DNA was identified the virus was also found in 14 swabs from cervix. There is a 34 times greater relative risk (OR = 34.446; CI: 10.194–116.353) of HPV DNA incidence in swabs from cervix if HPV DNA is found in oral mucosa smears of the patient. The correlation between HPV infections in the cervical/buccal smears of woman giving birth was presented in Table 3.

A well-documented relation between the frequency of HPV infections, young age, contraceptives intake, smoking, number of sexual partners, marital status, and level of education obtained exists [15, 21, 22]. A research team led by Takakuwa analysed a study group of 1183 pregnant patients among whom HPV infection was detected in 12.5% of cases [23]. It is worth noticing that young patients are statistically more prone to HPV infections, as HPV DNA was detected in 22.6% of the patients under the age of 25 and 11.3% of older patients (*p* < 0.0005). Place of residence of the patients might be another factor that determines the frequency of HPV infections. In pregnant and nonpregnant women in Uganda HPV DNA is detected in not less than 60% of cases, whereas it is estimated that 6.5% of pregnant women in Spain are infected [24, 25]. A research conducted in a group of 291 pregnant women in South Korea determined the frequency of HPV infection to be 18.9%. Additional stress was put on more frequent HPV infections in women with abnormal cytology with no record of pregnancies and under the age of 30 at the time of the tests [26]. Furthermore, a division between pregnant women without symptoms of infection and pregnant patients with visible changes to the skin and mucosa was made. In a group of pregnant patients suffering from an asymptomatic HPV infection Watts assessed the transmission of virus to newborns to be 1% [27]. Similar results can be found in research conducted by Kashima and coauthors who focused on a group of 231 newborns in whose swabs from mucosa and genital region HPV DNA was observed [28]. What is more, Smith and coauthors in their research on the incidence of HPV infection in children of infected mothers concluded that HPV DNA was found in 18% of swabs taken from 6-week-old children [29]. Presented data prove the existence of vertical HPV transmission, although the risk is considered low [18, 23, 30, 31]. However, the virus transmission incidence in cases of mothers who developed symptoms of infection is estimated to be 5% to 72% [32, 33]. Higher relative risk of HPV infection in child whose mother is HPV (+) seems to be logical and valid [16, 18, 27]. The dissimilarities between results obtained during medical research might be dependent on the number of copies of the virus in the assessed study group, and the employment of a variety of methods for HPV DNA detection [33–35]. One of the many factors of such correlation between the occurrences of HPV infection in the two analysed environments might be local defensive mechanisms. According to Smith et al. infections in swabs from cervical smears were 10 times more frequent than in swabs from buccal smears [14]. Similar trends have been noted in publications concerning pregnant women [36, 37]. Smaller number of detected HPV infections in buccal smears might be caused by different defensive mechanisms active in that particular environment. The microbiological protection of oral cavity and, more so, the saliva might be crucial to the defence against infection risk factors [38–40]. There were other study limitations. The studied group could be more numerous, due to little prevalence of infected participants. It is difficult to reliably verify the history of infections, because some might be unidentified. However, the results of the research work could be relevant in clinical practice. The analysis of the incidence of infection of human papillomavirus HPV in family environment showed an important and often overlooked significance of other test methods used for the identification of HPV infection. It seems that the introduction of a readily available and comfortable oral mucosa swab test should be much more promoted and encouraged. The identification of mothers in whose swabs of the oral mucosa HPV DNA was detected can help develop a group of children who run a relatively significant risk of being infected. Further studies may be needed to monitor the development of newborns infections.

## 4. Conclusions

A connection between buccal HPV incidence in mothers and newborns was proved in the research. A statistically significant difference in HPV DNA incidence in cervical smears in relation to HPV DNA occurrence in mothers' buccal smears was found. A thorough examination and follow-up of HPV (+) mothers' newborns may be vital for the selection of a group of children prone to HPV-related entities.

## Figures and Tables

**Table 1:** The correlation of the frequency of HPV infections between buccal smears (*n* = 152).

	Newborn buccal smears		Total number (%)^1^
	HPV DNA (+)	HPV DNA (−)	
Mother buccal smears HPV DNA (+)	8^*∗*^	11	19 (12.5%)
Mother buccal smears HPV DNA (−)	8	125	133 (87.5%)
Total number (%)^2^	16 (10.53%)	136 (89.47%)	152 (100%)

^1^Mother buccal smears.

^2^Newborn buccal smears.

^*∗*^ *p*<0.001.

**Table 2:** The correlation between HPV DNA incidence in oral mucosa of newborn and the cervix of mother (*n* = 152).

	Newborn buccal smears		Total number (%)^1^
	HPV DNA (+)	HPV DNA (−)	
Mother cervical smears HPV DNA (+)	11^*∗*^	13	24 (15.79%)
Mother cervical smears HPV DNA (−)	5	123	128 (84.21%)
Total number (%)^2^	16 (10.53%)	136 (89.47%)	152 (100%)

^1^Mother cervical smears.

^2^Newborn buccal smears.

^*∗*^ *p*<0.001.

**Table 3:** The correlation between HPV DNA incidence in the cervical/buccal smears of mothers (*n* = 152).

	Mother cervical smears		Total number (%)^1^
	HPV DNA (+)	HPV DNA (−)	
Mother buccal smears HPV DNA (+)	14^*∗*^	5	19 (12.5%)
Mother buccal smears HPV DNA (−)	10	123	133 (87.5%)
Total number (%)^2^	24 (15.57%)	128 (84.43%)	152 (100%)

^1^Mother buccal smears.

^2^Mother cervical smears.

^*∗*^ *p*<0.001.
